# Family-centered Cesarean Section for Placenta Accreta Spectrum: Questions and an Addition

**DOI:** 10.1055/s-0043-1770132

**Published:** 2023-06-20

**Authors:** Shigeki Matsubara

**Affiliations:** 1 Jichi Medical University, Tochigi, Japan

Dear Editor,

Nieto-Calvache et al. 1 showed that family-centered cesarean section (FCS) was possible in 53.8% of patients undergoing cesarean section (CS) for placenta accreta spectrum (PAS). Main concepts of FCS are: earlier skin-to-skin contact and cesarean delivery in a relaxed atmosphere. 2 The rationale of this study accords with this: even in PAS-CS/surgery, 1) FCS will enable earlier skin-to-skin contact, and 2) a “companion” in the surgery theater will lower the patient's stress during CS, possibly reducing the occurrence of posttraumatic stress disorder. I fully agree with the first point. Data showed that FCS enabled earlier skin-to-skin contact at CS in general. 2 Recommending earlier skin-to-skin contact even at PAS-CS is reasonable. Regarding the second point, I wish to ask two questions and make one addition.

The first question regards the meaning of “companion.” Nieto-Calvache et al. 1 state the importance of the presence of a “companion” in the surgical theater. Companion has various meanings: partner (husband), pregnant woman's mother (or a relative), doula, or other person. Does a companion mean a doula? A doula is a professional who takes care of a pregnant woman during pregnancy, labor, and postpartum. Their presence is considered to lower the delivering woman's stress, facilitating comfortable labor, and reducing delivery-related psychological trauma. 3 A doula, different from a partner or a relative, is a medical or obstetric professional, and thus having a doula in the surgical theater may cause less concerns of staff and anesthesiologists.

The second question regards the follow-up system of the corresponding mother and baby. Follow-up may be important in preventing, or the early detection of, posttraumatic stress disorder. I wish to know how women after PAS-CS are followed in Nieto-Calvache et al.'s institute. 1 In many Japanese institutes, obstetric nurses or midwives psychologically support mothers during CS: I believe that this is also performed in many other countries. In our institute, we allocate a nurse or midwife to a high-risk pregnant woman (such as a woman with PAS) on an individual basis. This nurse or midwife takes care of the corresponding woman during pregnancy, labor, and postpartum. In postpartum, through a telephone interview, the attending nurse or midwife checks the mother and baby's condition. If there are signs of psychological problems, they contact a health care center in the corresponding area, and support the woman and baby. In Japan, this system works in a similar manner to the doula-system.

Another consideration is a specific aspect of PAS-CS/surgery. Women with PAS are informed that PAS-CS/surgery may sometimes cause mortality. In an advanced cancer surgery, when a patient is informed of surgery-related mortality, one may refuse surgery, depending on the mortality rate. However, in PAS, this is not an option and may cause a marked stress. 4 5

Nieto-Calvache et al. 1 did not show that FCS at PAS-CS reduces the occurrence of posttraumatic stress disorder. They should not be blamed for this because the purpose of their study was to show that FCS can be performed even at PAS-CS. I believe that, theoretically, this system will reduce maternal psychological sequelae, and thus can be employed depending on the institutes' situation. When introducing this system, I believe that clarifying who the “companion” is may be important as the first step. Understanding the concept of the doula and/or attending obstetric nurse system may help doctors formulate a total-care and follow-up system for the PAS-mother and baby, the second step. Paying attention to stress unique to PAS-patients may be the third step for its establishment.

Lastly, regional anesthesia and stable vital signs (reduced blood loss) are prerequisites of FCS at PAS-CS. An excellent team like Nieto-Calvache et al. 1 made this possible. Less experienced teams should proceed with caution in a step-by-step manner.
